# Secondary Structure Characterization of Glucagon Products by Circular Dichroism and Nuclear Magnetic Resonance Spectroscopy

**DOI:** 10.3390/molecules27227805

**Published:** 2022-11-12

**Authors:** Zhongli Bao, Ya-Chi Cheng, Justin Jun Wei, Mary Ziping Luo, Jack Yongfeng Zhang

**Affiliations:** Amphastar Pharmaceuticals, Inc., Rancho Cucamonga, CA 91730, USA

**Keywords:** glucagon, circular dichroism, nuclear magnetic resonance, spectroscopy, synthetic peptide, injection, CD, NMR, 2D NOESY, secondary structure, alpha helix

## Abstract

Glucagon, a 29-amino acid polypeptide hormone, is an essential therapeutic agent used in the emergency treatment of hypoglycemia. However, glucagon is inherently unstable in aqueous solution. While glucagon equilibrates between unordered and the secondary α-helix state in solution, it can quickly transform into a different secondary β-sheet-rich amyloid-like fibril/oligomer structure under various conditions. Since changes in the secondary structure of glucagon can cause significant impacts, structure analysis is necessary and essential to assess the safety of the product. This study analyzed the secondary structure of glucagon products at the release and at the expiry using circular dichroism spectroscopy (CD) and 2D Nuclear Overhauser effect spectroscopy (2D NOESY). In order to also determine if structural differences exist between glucagon produced through different manufacturing processes, synthetic and recombinant glucagon products were used and compared. The CD results indicated that for all release and expired glucagon products, the structure compositions were 14 to 16% α-helix, 17 to 19% β-strand, 14 to 15% Turn, and 53 to 54% Unordered. This was consistent with the 2D NOESY analysis which showed that both products had an approximate α-helix composition of 14 to 17%. Overall, there were no significant differences in terms of the secondary structure between synthetic and recombinant glucagon products both at the release and at the expiry.

## 1. Introduction

Glucagon, a 29-amino acid polypeptide hormone, is an essential therapeutic agent used in the emergency treatment of hypoglycemia [1]. However, glucagon is inherently unstable in aqueous solution. While glucagon equilibrates between unordered and the secondary alpha-helix state in solution, it can quickly transform into a different secondary β-sheet-rich amyloid-like fibril/oligomer structure under various conditions such as a different concentration, pH, temperature, and time [2]. The formation of fibrillary aggregates is a serious type of degradation in glucagon as it can occur rapidly, which may affect the drug potency and increase the risk of life-threatening immunogenicity [3]. As a result, commercial glucagon products are typically formulated as lyophilized powder to be reconstituted with diluent for an immediate use [4]. Recently, a synthetic version of glucagon for injection was approved by the FDA as a generic to Eli Lilly’s Glucagon Emergency Kit, which is of a recombinant DNA origin [5]. Briefly, the recombinant glucagon is produced in a laboratory strain of *Escherichia coli* bacteria that has been genetically altered by the addition of the gene for glucagon [6]. In contrast, the synthetic glucagon is chemically synthesized using a solid-phase peptide synthesis (SPPS) method. SPPS involves stepwise additions of protected amino acid derivatives to a growing peptide chain, followed by cleavage from the resin, including cleavage of the remaining protecting groups, pre-purification, and purification steps by preparative high-performance liquid chromatography [7,8].

Since any changes in the secondary structure of a protein or peptide such as glucagon can cause a significant impact on its function, it is of interest to evaluate the secondary structural characteristics of synthetic glucagon in comparison to the recombinant version. Circular dichroism (CD) [9,10] and nuclear magnetic resonance (NMR) spectroscopy [11] are two common techniques used to study the structure of glucagon in solution.

Circular dichroism is increasingly recognized as a valuable technique for examining the structure of proteins in solution [12,13,14,15,16]. Briefly, the method is based on the differential absorption of left and right circularly polarized light. When circularly polarized light passes through the medium, an optically active chiral compound will absorb the left and right polarized light by different amounts. The difference in the left- and right-handed absorbance is the signal registered in the CD spectra. The ordered α-helices, β-sheets, β-turn, and random coil conformations all have characteristic spectra. These unique spectra form the basis for protein secondary structure analysis [16].

Nuclear magnetic resonance spectroscopy (NMR) is another technique often used to provide the structural and dynamic characterization of peptides and proteins [11,17,18,19]. Structure determination by NMR spectroscopy consists of several phases, each using a separate set of highly specialized techniques [18]. There are three 2D spectra which are widely used for the structure determination of proteins with a mass of up to 10kD: 2D correlation spectroscopy (COSY) and 2D total correlation spectroscopy (TOCSY) experiments which identify amino acid spin system and 2D nuclear Overhauser effect spectroscopy (NOESY) which identifies neighboring amino acids and sequentially connects the amino acid spin systems. While TOCSY only provides information for self cross-peaks (NH_i_ and αH_i_), 2D NOESY shows both self (NH_i_-αH_i_) and sequential cross-peaks (NH_i_-αH_i-1_) in the same region. Therefore, by overlapping the spectrums produced from TOCSY and NOESY, the αH groups of the amino acid can be located. The connectivity of this amino acid residue (i-1) to its following amino acid residue (i) can be observed in NOESY, which can further identify its neighboring amino acid residue [19]

Previous literatures have successfully observed the secondary structure of glucagon under various conditions, with the α-helical region being in the Phe^22^ to Leu^26^ fragment [9,10,11]. However, there were no studies evaluating and comparing the secondary structure of synthetic and recombinant glucagon products. This study provides a structural comparison of synthetic and recombinant glucagon through a combination of the commonly used techniques of circular dichroism (CD) and nuclear magnetic resonance spectroscopy (NMR).

## 2. Results

### 2.1. Secondary Structure Evaluation by CD Spectroscopy

**Figure 1:**
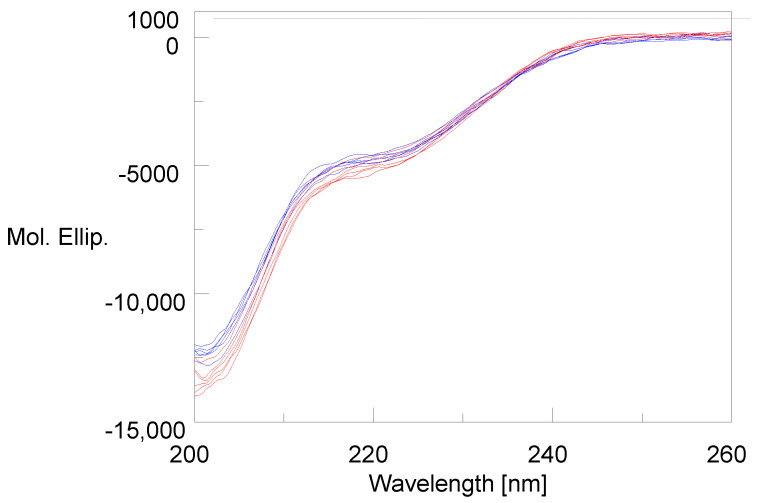
The CD spectra show the structures of synthetic glucagon and recombinant glucagon (new or aged lots) are equivalent. Three lots of AMP-glucagon at release and three lots at expiry (colored blue), three lots of recently manufactured and three lots of expired ELI-glucagon (colored red).

The CD spectra of glucagon are shown in Figure 1, which includes three lots of AMP-glucagon at the release and three lots at expiry (colored blue), three lots of recently manufactured, and three lots of expired ELI-glucagon (colored red). The spectra were truncated to 200–260 nm to remove the noise in the 185–200 nm range. The CD spectra in Figure 1 show that both synthetic and recombinant glucagon have very similar spectra with no differences observed between the recent and aged lots.

The secondary structure percentage obtained through the CD spectra for both at the release and at the expiration of AMP-glucagon and ELI-glucagon are summarized in Table 1. For the AMP-glucagon solution, the CD results showed about 14 to 16% α-helix, 17 to 19% β-strand, 14 to 15% Turn, and 53 to 54% Unordered at the release and expiration. The ELI-glucagon showed very similar results with 14 to 17% α-helix, 15 to 17% β-strand, 14 to 15% Turn, and 53 to 55% Unordered for the recently manufactured and expired lots.

Based on the CD data in Table 1, the ELI-glucagon extremes were obtained and the equivalence evaluation criteria (EEC) limits were determined using the established EEC equation. The EEC for the secondary structure data of glucagon at the release and at the expiration are summarized in Table 2. It is demonstrated that the secondary structure (α-helix, β-sheet, turn, and unordered) of AMP-glucagon meets the EEC at both the release and at the expiration.

To further confirm the study’s results, two additional CD data analysis programs (SELCON3 and CDSSTR) were used to estimate the secondary structure of glucagon. While the %helix content from CONTIN spans from 13.8 to 17.1, it appears to be slightly lower using SELCON3 (12.1–13.5) and CDSSTR (9.4–11.2). The CD results from all three programs indicate an α-helix structure in glucagon having approximately 3–5 amino acids.

### 2.2. Structure Characterization of Glucagon by NMR

**Figure 2:**
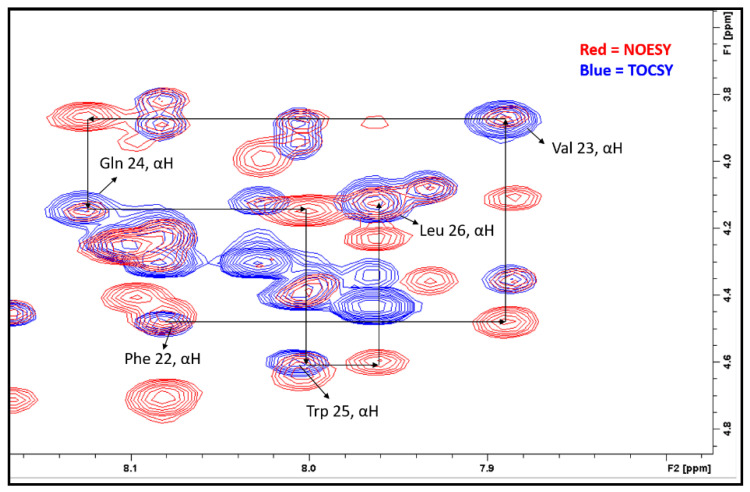
TOCSY and NOESY overlaid spectra demonstrates the sequential walk for the α-H on Phe^22^Val^23^Gln^24^Trp^25^Leu^26^.

The TOCSY and NOESY sequential walk method was used for the identification and sequential assignment of the amino acid residues [19,20,21]. In this method, NOESY and TOCSY spectra were acquired separately and then overlaid, as shown in Figure 2. The TOCSY spectrum (blue) only showed self cross-peaks (NH_i_ and αH_i_) while the NOESY spectrum (red) showed both self (NH_i_-αH_i_) and sequential (NH_i_-αH_i-1_) cross-peaks in the fingerprint region. The peaks appearing in both the NOESY and TOCSY spectrums are αH groups of the amino acid. The connectivity of this given amino acid residue (i-1) to its following residue (i) can be observed in NOESY. Using sequential walking, the cross-peaks of the amino acids Phe^22^, Val^23^, Gln^24^, Trp^25^, and Leu^26^ were identified from hundreds of other cross-peaks in the NOESY spectrum.

**Figure 3:**
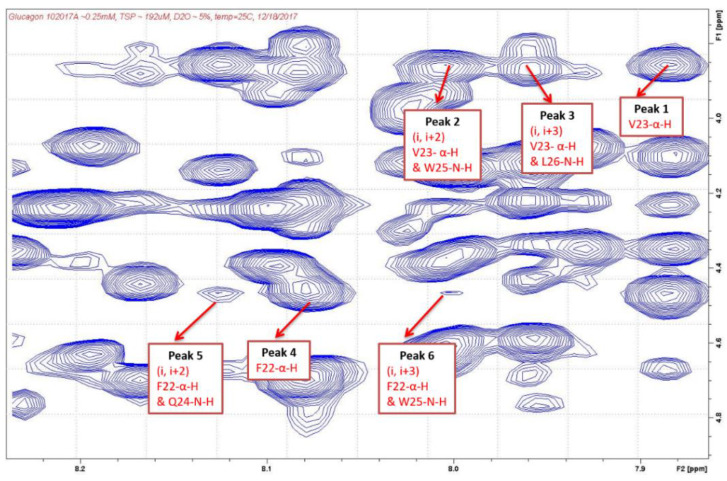
NOESY cross-peaks in AMP-glucagon.

**Figure 4:**
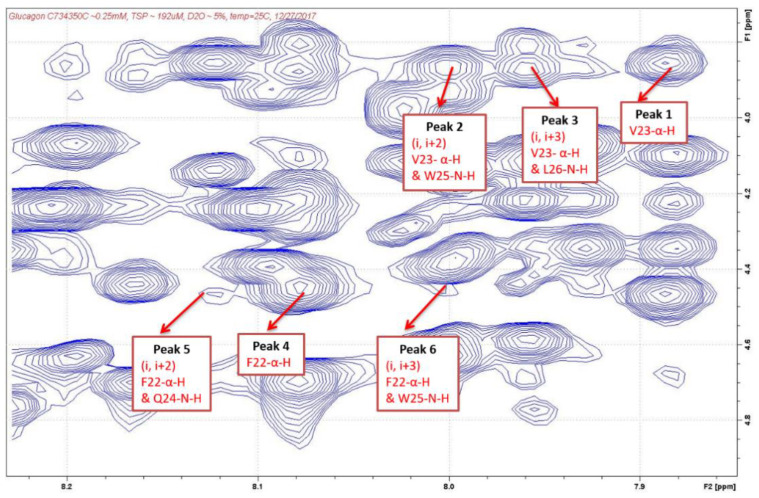
NOESY cross-peaks in ELI-glucagon.

After the cross-peaks of Phe^22^, Val^23^, Gln^24^, Trp^25^, and Leu^26^ were identified, the interactions between the α-H and N-H of these amino acids were determined which indicated the presence of a helix structure. As shown in Table 3 and Figure 3 and Figure 4, strong intensity dαN(*i*, *i*+2) and dαN(*i*, *i*+3) cross-peaks were observed between the αH of Val^23^ and the NH of Trp^25^ and NH of Leu^26^ for both AMP-glucagon and ELI-glucagon, both at the release and at the end of the shelf life. In addition, weak intensity dαN(*i*, *i*+2) and dαN(*i*, *i*+3) cross-peaks were observed between the α-H of Phe^22^ and the NH of Gln^24^ and the NH of Trp^25^ for all the glucagon products. This result indicates that all the reconstituted glucagon products have a local non-random spatial structure spanning residues of Phe^22^-Val^23^-Gln^24^-Trp^25^-Leu^26^.

The complete chemical shift assignments of ELI-glucagon and AMP-glucagon are provided in Tables S1 and S2, respectively. For both glucagon products, the majority of the glucagon residues were assigned in the fingerprint region, with a few residues ambiguously assigned due to overlapping peaks and weak peaks such as the α-H of Lys^12^. The observed chemical shifts were then compared with the respective reference values based on the chemical shift index. The Hα chemical shifts of Phe^22^ to Leu^26^ for both ELI-glucagon (Table S3 and Figure S1) and AMP-glucagon (Table S4) were shown to be less than the CSI reference values (≤0.1 ppm or CSI of −1), which indicates an α-helix between Phe^22^ and Leu^26^. It was noted that several residues such as Ser^11^ and Met^27^ had possible alpha helix regions with CSI index values of −1. These residues were further analyzed for dαN connectivity, however, these residues did not have dαN(*i*, *i*+2) and/or dαN(*i*, *i*+3) interactions, indicating that there was no alpha helix. The dαN connectivities were further confirmed by dNN connectivity studies which show Phe^22^ and Leu^26^ amide proton connectivities [dNN(*i*, *i*+1), (*i*, *i*+2), (*i*, *i*+3), and (*i*, *i*+4)] in both ELI-glucagon and AMP-glucagon (Figures S2–S4).

To confirm the sequential assignment, the COSY spectra were also measured (provided in Supplementary Material). The COSY spectrum in Figure S5 shows a cross-peak between the NH (7.89 ppm) and αH (3.85 ppm) of Val^23^, confirming the NH and αH assignment from the NOESY and TOCSY sequential assignment method. In addition, the COSY spectrum in Figure S6 shows cross-peaks from αH-βH and βH-ƴH interactions, which further confirms the αH, βH, and ƴH assignment of Val^23^.

Based on the NMR results, it is likely that there is a one turn alpha helix structure in the sequence of Val^23^-Gln^24^-Trp^25^-Leu^26^ (four residues), Phe^22^-Val^23^-Gln^24^-Trp^25^ (four residues), or Phe^22^-Val^23^-Gln^24^-Trp^25^-Leu^26^ (five residues) for both glucagon products at both the release and at the expiration. The results suggests that there are likely four–five amino acids which form an α-helix. Since glucagon contains 29 amino acids, 14% (4/29) to 17% (5/29) are calculated to be the α-helix.

## 3. Discussion and Conclusions

The results of this circular dichroism study demonstrated an equivalency in terms of the secondary structure between a synthetic (AMP-glucagon) and recombinant (ELI-glucagon) glucagon product. Interestingly, the CD results showed no significant differences between the two products for the secondary ordered structures including an alpha helix, beta strand, turn, and unordered structure. In addition, for all the tested lots at the release and at the expiry, there were no significant differences in the secondary structure. Further, the alpha helix percentage found in this study (14–16%) is also consistent with previous studies in the literature which found dilute glucagon solutions (<1 mg/mL) to have an α-helix composition of 10–15% [9].

The results of the 2D NMR analysis were also similar to that of the CD, with approximately 14–17% of the glucagon having an alpha helix structure for both synthetic and recombinant glucagon products. In general, an α-helix consists of 3.6 amino acids per helical turn, and is characterized by NOE cross-peaks between residues dαN(*i*, *i*+2), dαN(*i*, *i*+3), and dαN(*i*, *i*+4) [22,23,24,25]. Our findings indicated that this turn occurs at a specific sequence, Phe^22^-Val^23^-Gln^24^-Trp^25^-Leu^26^, in both of the glucagon products tested.

Previous ^1^H NMR studies demonstrated that glucagon has an α-helical structure [11,26] with Phe^22^-Leu^26^ having a local non-random α-helical structure [11], which is essential for receptor binding [27]. Since the Phe^22^-Leu^26^ peptide fragment has such an important function in the biological activity of glucagon, a further 2D NMR structure study using NOESY and TOCSY on Phe^22^-Leu^26^ may be valuable. However, previous 2D NMR studies mainly assessed glucagon bound to lipid micelles glucagon [28], glucagon amyloid fibril [29] or glucagon analogs (desHis^1^, desPhe^6^, and Glu^9^) [30], and GLP-1 [31]. Further, there were no 2D NMR structural studies on commercial glucagon. Our study expanded on previous findings by evaluating and comparing the secondary structure of commercial synthetic and recombinant glucagon products using 2D NMR and circular dichroism (CD).

Overall, the CD and NMR structural findings of this study indicated that the synthetic and recombinant glucagon products are similar with no major differences in the secondary structure throughout the entirety of both shelf lives.

## 4. Materials and Methods

Glucagon samples of different manufacturing dates (i.e., recently released and expired lots) were obtained and studied. Six lots of synthetic glucagon were provided by Amphastar Pharmaceuticals, Inc. (Rancho Cucamonga, CA, USA), of which three lots were recently manufactured and three were expired lots. Six lots of recombinant glucagon (Eli Lilly and Company, Indianapolis, IN, USA) were purchased commercially and used as the reference listed drug (RLD): three lots were recently purchased, and three were expired. The expired lots were studied to determine whether there were any significant changes in the secondary structure of glucagon products after long-term storage. In this study, the synthetic glucagon is referred to as AMP-glucagon and the RLD recombinant glucagon is referred to as ELI-glucagon.

### 4.1. Circular Dichroism Study

For the quantitative characterization of the secondary structure through the CD analysis for glucagon in solution, it is important to maintain a specific condition at which the secondary structure of glucagon is stable. In this characterization study, the following conditions were utilized: a glucagon concentration of 0.25 mg/mL, a solution pH at 2, a temperature at around 20 °C, a diluent (12 mg/mL of glycerin, pH 2.0), and only fresh samples were used. For each sample preparation, 1 mL of glucagon was reconstituted with 3 mL of diluent in a vial.

CD experiments were performed on a JASCO J-815 (Hachioji, Tokyo, Japan) spectrometer equipped with a Peltier type cell holder. The spectra were recorded at a temperature of 20 °C, a scanning speed of 5 nm/min, and a band width of 1 nm. To calculate the secondary structure components (percent of α-helix, β-strand, turn, and unordered) of the glucagon, the measured CD spectra were smoothed with a mean window of 25 data points and truncated to 200–260 nm to remove the noise in the range of 185–200 nm [2,14]. The per-residue molar protein concentration and cuvette path length of 0.1 cm were entered in the CDPro analysis program, and the SP37A soluble protein CD database and CONTIN fit of the data were applied.

In order to evaluate whether the secondary structure of the AMP-glucagon is equivalent to that of the ELI-glucagon, the equivalence evaluation criteria (EEC) was determined. The EEC was established based on the following equations:

EEC Lower Limit = *R_min_* · (1 − *η*) (1)

EEC Upper Limit = *R_max_* · (1 + *η*) (2)

where *R_min_* and *R_max_* are the minimum and maximum (overall extreme) of the results for the ELI-glucagon lots, i.e., the lowest value and the highest value, respectively, for the tested characterization parameters at a specific time: (i) recently manufactured ELI-glucagon or (ii) expired ELI-glucagon. *η* is the allowed percentage range, which is 10% for the CD analysis for the secondary structure of glucagon. The secondary structure data (percent of α-helix, β-strand, turn, and unordered) of the synthetic glucagon is considered to be equivalent if the value falls within the EEC limits.

### 4.2. Nuclear Magnetic Resonance (NMR) Study

The solution used for the reconstituted glucagon sample consisted of 990 µL of original diluent, 8 µL of 25 mM Trimethylsilylpropanoic acid (TSP-d4) in D_2_O, and 42 µL of D_2_O. A 2D TOCSY was conducted to identify the amino acid spin system. The 2D TOCSY was conducted on one lot of ELI-glucagon and one lot of AMP-glucagon, and the spectra were acquired on a 700 MHZ NMR using a modified sequence to suppress the peaks of water, lactose, and glycerin. The mixing time of the 2D TOCSY was 60 ms, which provides a spectrum that almost shows the whole amino acid spin system.

Next, the 2D-NOESY was conducted to identify the neighboring amino acid residues and sequentially connect the amino acid spin systems. The NMR data were acquired on a 700 MHz Bruker instrument equipped with a cryoprobe. The mixing time of the 2D ^1^H-^1^H NOESY was 250 ms. After the NOESY mixing period, one shaped 90 pulse and one shaped 180 pulse were used which only excited the amide regions, so that no lactose, glycerin, or water signals would be detected. The acquisition time on the direct and indirect dimensions were about 104 ms and 30.4 ms, respectively. The NMR measurements were performed at 300 K. The spectra were processed with the Bruker Biospin TopSpin 2.1.1 software (Rheinstetten, Germany).

The spectra from NOESY and TOCSY were then overlaid to locate the *α*H groups of the amino acid. While the TOCSY spectrum only gives NH_i_ and αH_i_ self cross-peaks in the fingerprint region, NOESY fingerprint gives both self (NH_i_-αH_i_) and sequential (NH_i_-αH_i-1_) cross-peaks in the same region. Therefore, peaks that appeared in both the NOESY and TOCSY spectrums are the αH groups of the amino acid in the fingerprint region. The neighboring amino acids in the sequence can be further identified. In addition, a 2D COSY was also performed to confirm the sequential assignment results.

## Figures and Tables

**Table 1:** Secondary Structure Content of AMP-Glucagon and ELI-Glucagon by CD.

Products	Time for Shelf Life	Lot Number	Secondary Structure Tested by CD			
			α Helix %	β Strand %	Turn %	Unordered %
AMP-glucagon	At release	102017	14.4	17.5	14.6	53.6
		102017A	13.9	17.4	14.6	54.1
		102017B	13.8	17.9	14.3	54.1
ELI-glucagon	Recently manufactured	C304844A	14.3	17.4	14.6	53.7
		C304844C	14.1	17.3	14.6	53.9
		C283822C	14.4	17.3	14.6	53.8
AMP-glucagon	Expired > 3 years	21914	14.3	18.2	14.4	53.0
		021914A	13.9	18.5	14.5	53.2
		021914B	15.8	16.9	14.1	53.1
ELI-glucagon	End of shelf life	C494099D	16.7	15.4	14	53.9
		C505143A	17.1	16.8	13.6	52.6
		C502399D	14.1	16.7	14.5	54.8

**Table 2:** Equivalence Evaluation for Secondary Structure with CD Method for AMP-Glucagon vs. ELI-Glucagon.

Equivalence Evaluation Type	Establishment of EEC & Equivalence Evaluation			Secondary Structure, Tested by CD			
				α Helix (%)	β Strand (%)	Turn(%)	Unordered (%)
AMP-glucagon vs. ELI-glucagon **at release**	Extreme of ELI-glucagon, fresh lots (n = 3)		R_Min_	14.1	17.3	14.6	53.7
			R_Max_	14.4	17.4	14.6	53.9
	EEC at release *		Lower	12.7	15.6	13.1	48.3
			Upper	15.8	19.1	16.1	59.3
	AMP-glucagon meets EEC?	AMP-glucagon lot No.	102017	√	√	√	√
			102017A	√	√	√	√
			102017B	√	√	√	√
AMP-glucagon vs. ELI-glucagon **at expiration**	Extreme of ELI-glucagon, Expired lots (n = 3)		R_Min_	14.1	15.4	13.6	52.6
			R_Max_	17.1	16.8	14.5	54.8
	EEC at expiration *		Lower	12.7	13.9	12.2	47.3
			Upper	18.8	18.5	16.0	60.3
	AMP-glucagon meets EEC?	AMP-glucagon lot No.	21914	√	√	√	√
			021914A	√	√	√	√
			021914B	√	√	√	√

* EEC = ELI-glucagon extreme ± 10%, i.e., lower limit = 0.9 × R_Min_, and upper limit =1.1 × R_Max._

**Table 3:** NOESY Cross-Peak Intensity for AMP-Glucagon and ELI-Glucagon.

Glucagon Amino Acid Sequence			Val^23^-Gln^24^-Trp^25^-Leu^26^			Phe^22^-Val^23^-Gln^24^-Trp^25^		
Amino acid residue			Val^23^	Trp^25^	Leu^26^	Phe^22^	Gln^24^	Trp^25^
			αH	NH	NH	αH	NH	NH
			*i*	*i*+2	*i*+3	*i*	*i*+2	*i*+3
NOE cross-peak chemical shift (ppm)		N-H	7.89	8.00	7.96	8.08	8.12	8.00
		α-H	3.85	3.85	3.85	4.45	4.45	4.45
NOESY cross-peak intensity (abs, 10^5^)	AMP-glucagon (at release)	102017	-	2.1	1.2	-	0.3	0.2
		102017A	-	2.0	1.2	-	0.3	0.2
		102017B	-	1.9	1.2	-	0.3	0.2
	ELI-glucagon (recently manufactured)	C734350C	-	1.5	0.9	-	0.2	Visible
		C699511C	-	1.4	0.8	-	Visible	0.2
		C753564A	-	1.7	0.9	-	0.2	Visible
	AMP-glucagon (expired)	21914	-	1.6	0.9	-	0.2	0.2
		021914A	-	2.0	1.3	-	0.3	0.2
		021914B	-	1.7	1.1	-	0.3	0.2
	ELI-glucagon (expired)	C505143A	-	1.6	0.9	-	0.2	0.2
		C502399D	-	1.8	1.0	-	0.2	Visible
		C404099D	-	1.7	0.9	-	0.2	0.2

## Data Availability

Not applicable.

## Supplementary Materials

The following supporting information can be downloaded at https://www.mdpi.com/article/10.3390/molecules27227805/s1, Figure S1: The Chemical shift index analysis of ELI-Glucagon; Figure S2: F22 Amide Proton connectivity [dNN (*i*, *i*+1), (*i*, *i*+2), (*i*, *i*+3), (*i*, *i*+4)] (ELI-Glucagon); Figure S3: V23 Amide Proton connectivity [dNN (*i*, *i*+1), (*i*, *i*+2), (*i*, *i*+3), (*i*, *i*+4)] (ELI-Glucagon); Figure S4: F22 Amide Proton connectivity [dNN (*i*, *i*+1), (*i*, *i*+2), (*i*, *i*+3), (*i*, *i*+4)] (AMP-Glucagon); Figure S5: COSY image of glucagon (NH-αH Region); Figure S6: COSY image of glucagon (αH-βH-ƴH Region); Table S1: ^1^H Chemical Shift Assignment (PPM) of ELI-Glucagon using 2D NMR; Table S2: ^1^H Chemical Shift Assignment (PPM) of AMP-Glucagon using 2D NMR; Table S3: The Chemical shift index analysis of ELI-Glucagon; Table S4: The Chemical shift index analysis of AMP-Glucagon.
