# Supplementary material for: Secondary Structure Characterization of Glucagon Products by Circular Dichroism and Nuclear Magnetic Resonance Spectroscopy
Source: Molecules. 2022 Nov 12;27(22):7805. doi: 10.3390/molecules27227805 (PMC9696263; doi:10.3390/molecules27227805)
Supplement: Supplementary file 1 [file molecules-27-07805-s001.zip › molecules-1960395-supplementary.pdf]

Supplementary Information

Secondary Structure Characterization of Glucagon Products by Circular Dichroism and Nuclear Magnetic Resonance Spectroscopy

Zhongli Bao, Ya-Chi Cheng, Justin Jun Wei, Mary Ziping Luo, and Jack Yongfeng Zhang*

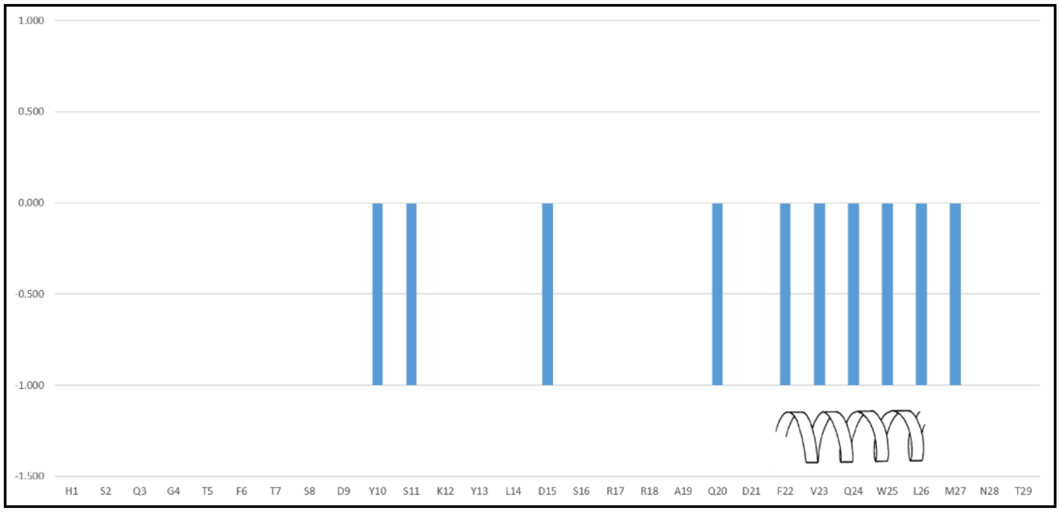

Figure S1. The Chemical shift index analysis of ELI-Glucagon

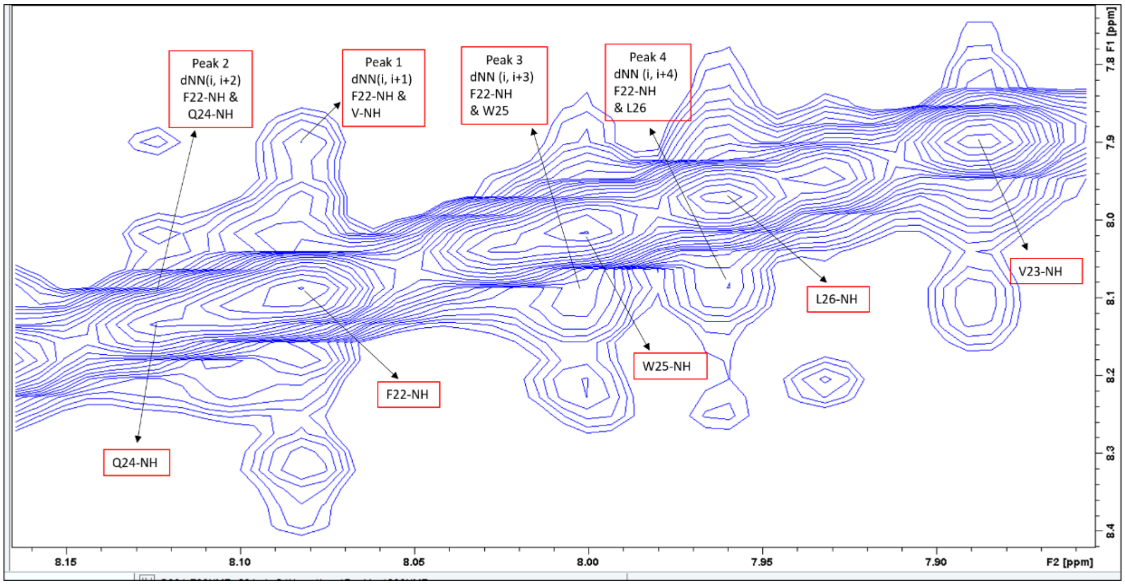

Figure S2. F22 amide proton connectivity [dNN(*i*, *i*+1), (*i*, *i*+2), (*i*, *i*+3), (*i*, *i*+4)] (ELI-Glucagon)

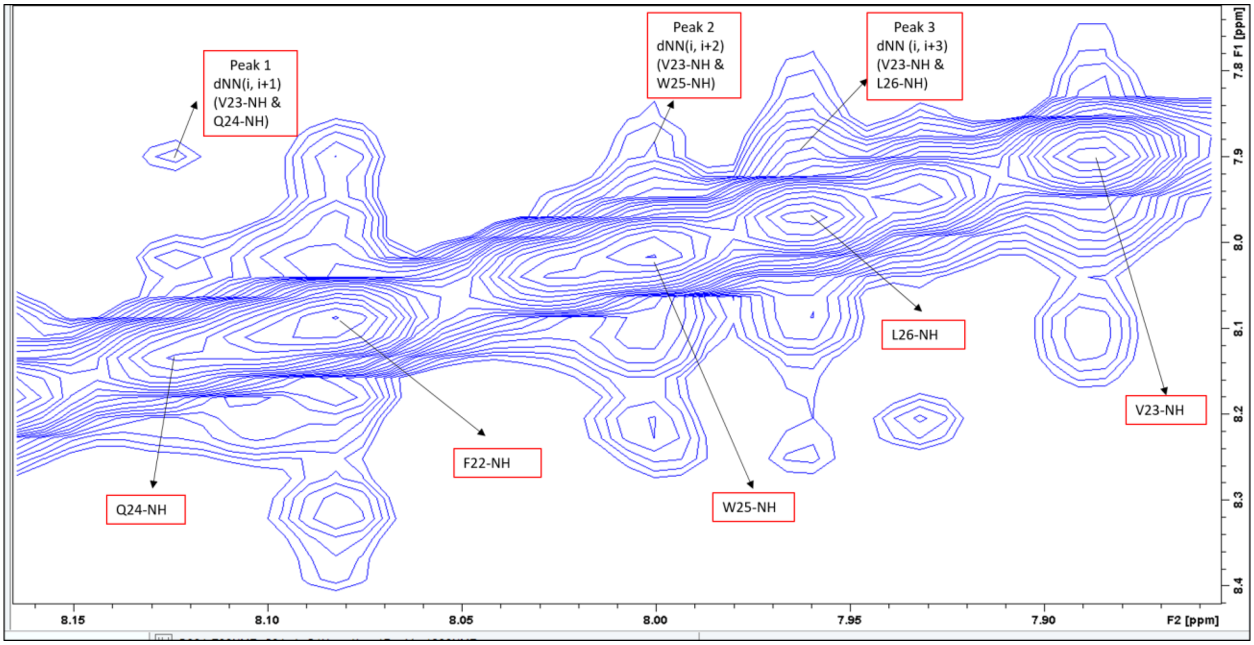

Figure S3. V23 amide proton connectivity [dNN(*i*, *i*+1), (*i*, *i*+2), (*i*, *i*+3), (*i*, *i*+4)] (ELI-Glucagon)

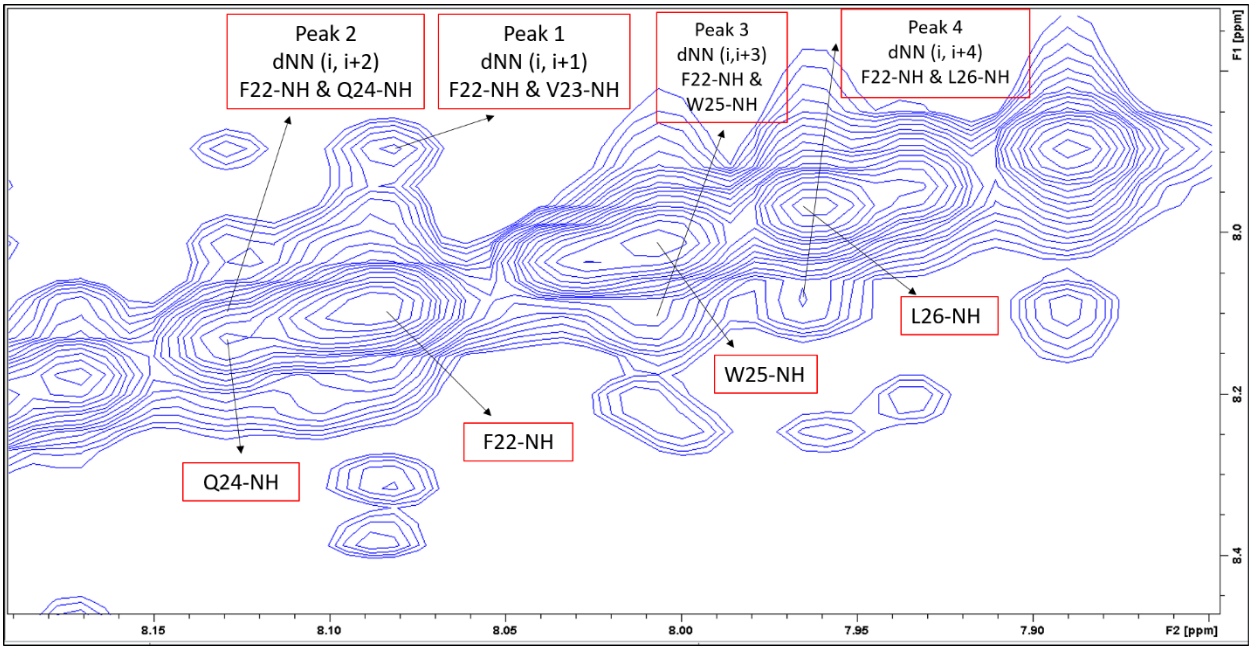

Figure S4. F22 amide proton connectivity [dNN(*i*, *i*+1), (*i*, *i*+2), (*i*, *i*+3), (*i*, *i*+4)] (AMP-Glucagon)

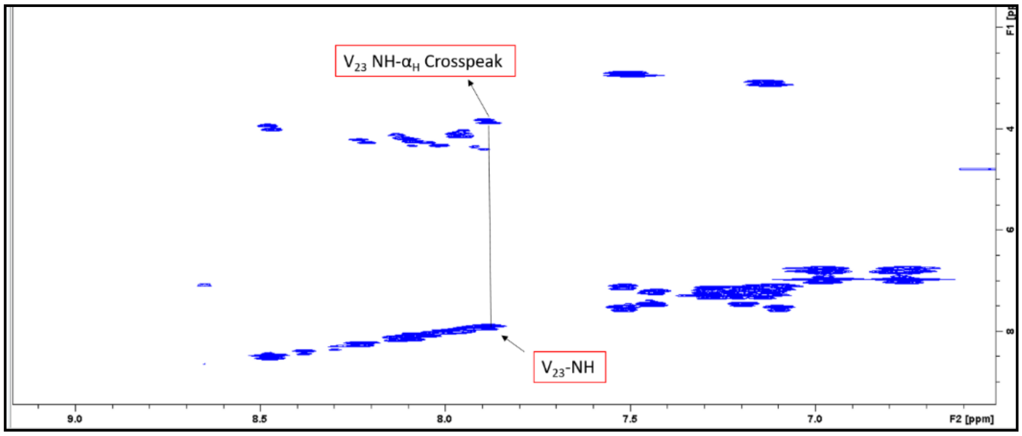

Figure S5. COSY image of glucagon (NH-αH Region)

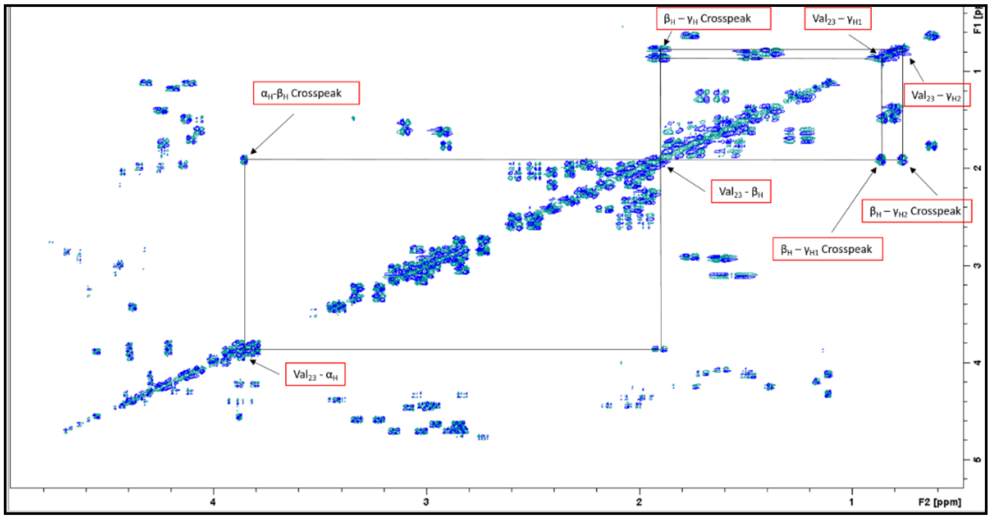

Figure S6. COSY image of glucagon (αH-βH-γH Region)

Table S1. ^{1}H Chemical Shift Assignment (PPM) of ELI-Glucagon using 2D NMR Methods

Residue	NH (PPM)	$C^{\alpha}H$ (PPM)	$C^{\beta}H$ (PPM)	Others (PPM)
His 1	n.o.*	n.o.*	n.o.*	
Ser 2	8.86	4.55	3.88	
Gln 3	8.75	4.42	2.01, 2.15	$C^{\gamma}H$ = 2.42
Gly 4	8.46	3.98		
Thr 5	8.02	4.30	4.11	$C^{\gamma}H$ = 1.10
Phe 6	8.37	4.70	3.01, 3.15	
Thr 7	8.08	4.29	4.20	$C^{\gamma}H$ = 1.17
Ser 8	8.29	4.41	3.91, 3.84	
Asp 9	8.47	4.69	2.86	
Tyr 10	8.16	4.44	2.98	
Ser 11	8.08	4.23	3.82, 3.87	
Lys 12[a]	7.49 [a]	n.o.*	1.61 [a]	$C^{\gamma}H$ = 1.26; $C^{\delta}H$=1.62; $C^{\varepsilon}H$=2.92 [a]
Tyr 13[a]	7.88 [a]	4.36[a]	2.84, 3.05 [a]	
Leu 14	7.93	4.08	1.69	$C^{\gamma}H$ = 1.47; $C^{\delta}H$=0.82
Asp 15	8.20	4.65	2.85, 2.95	
Ser 16	8.00	4.41	3.87, 3.92	
Arg 17[a]	7.12[a]	4.24[a]	1.74, 1.82 [a]	$C^{\gamma}H$ = 1.58; $C^{\delta}H$=3.10 [a]
Arg 18[a]	8.10[a]	4.26[a]	1.74, 1.82 [a]	$C^{\gamma}H$ = 1.58; $C^{\delta}H$=3.10 [a]
Ala 19[a]	8.21[a]	4.23[a]	1.40 [a]	
Gln 20	8.22	4.25	1.95, 2.02	$C^{\gamma}H$ = 2.32
Asp 21	8.31	4.68	2.99	
Phe 22	8.08	4.45	3.07, 2.99	
Val 23	7.89	3.85	1.89	$C^{\gamma}H$ = 0.84; 0.74
Gln 24	8.12	4.15	1.94	$C^{\gamma}H$ = 2.24
Trp 25	8.00	4.58	3.31, 3.21	
Leu 26	7.96	4.10	1.68	$C^{\gamma}H$ = 1.26; $C^{\delta}H$=0.81
Met 27	8.00	4.35	1.96, 2.06	$C^{\gamma}H$ = 2.50; 2.58
Asn 28	8.24	4.75	2.84, 2.75	
Thr29	7.96	4.40	4.11	$C^{\gamma}H$ = 1.10

n.o.* = not observed; [a] = ambiguously assigned

Table S2. ^{1}H Chemical Shift Assignment (PPM) of AMP-Glucagon using 2D NMR Methods

Residue	NH (PPM)	$C^{\alpha}H$ (PPM)	$C^{\beta}H$ (PPM)	Others (PPM)
His 1	n.o.*	n.o.*	n.o.*	
Ser 2	8.86	4.55	3.88	
Gln 3	8.75	4.43	2.01, 2.15	$C^{\gamma}H$ = 2.42
Gly 4	8.46	3.98		
Thr 5	8.02	4.31	4.11	$C^{\gamma}H$ = 1.10
Phe 6	8.37	4.70	3.01, 3.15	
Thr 7	8.08	4.29	4.20	$C^{\gamma}H$ = 1.17
Ser 8	8.29	4.41	3.91, 3.84	
Asp 9	8.47	4.69	2.86	
Tyr 10	8.17	4.45	2.98	
Ser 11	8.08	4.23	3.82, 3.87	
Lys 12[a]	7.49 [a]	n.o.*	1.61 [a]	$C^{\gamma}H$ = 1.26; $C^{\delta}H$=1.62; $C^{\varepsilon}H$=2.92 [a]
Tyr 13[a]	7.88 [a]	4.36[a]	2.84, 3.05 [a]	
Leu 14	7.93	4.08	1.69	$C^{\gamma}H$ = 1.47; $C^{\delta}H$=0.82
Asp 15	8.20	4.65	2.85, 2.95	
Ser 16	8.00	4.41	3.87, 3.92	
Arg 17[a]	7.12[a]	4.24[a]	1.74, 1.82 [a]	$C^{\gamma}H$ = 1.58; $C^{\delta}H$=3.10 [a]
Arg 18[a]	8.10[a]	4.26[a]	1.74, 1.82 [a]	$C^{\gamma}H$ = 1.58; $C^{\delta}H$=3.10 [a]
Ala 19[a]	8.21[a]	4.23[a]	1.40 [a]	
Gln 20	8.22	4.25	1.95, 2.02	$C^{\gamma}H$ = 2.32
Asp 21	8.31	4.68	2.99	
Phe 22	8.08	4.45	3.07, 2.99	
Val 23	7.89	3.85	1.89	$C^{\gamma}H$ = 0.84; 0.74
Gln 24	8.12	4.15	1.94	$C^{\gamma}H$ = 2.24
Trp 25	8.00	4.58	3.31, 3.21	
Leu 26	7.96	4.10	1.68	$C^{\gamma}H$ = 1.26; $C^{\delta}H$=0.81
Met 27	8.00	4.35	1.96, 2.06	$C^{\gamma}H$ = 2.50; 2.58
Asn 28	8.24	4.75	2.84, 2.75	
Thr29	7.96	4.40	4.11	$C^{\gamma}H$ = 1.10

n.o.* = not observed because of exchange; [a] = ambiguously assigned

Table S3. The Chemical shift index analysis of ELI-Glucagon

Residue	Real $C^{\alpha}H$ (PPM)	Reference $C^{\alpha}H$ (PPM)	Δ (Real $C^{\alpha}H$-Reference $C^{\alpha}H$) (PPM)	Index Value
S2	4.55	4.50	0.05	0
Q3	4.42	4.37	0.05	0
G4	3.98	3.97	0.01	0
T5	4.30	4.35	-0.05	0
F6	4.70	4.66	0.04	0
T7	4.29	4.35	-0.06	0
S8	4.41	4.50	-0.09	0
D9	4.69	4.76	-0.07	0
Y10	4.44	4.60	-0.16	-1
S11	4.23	4.50	-0.27	-1
L14	4.08	4.17	-0.09	0
D15	4.65	4.76	-0.11	-1
S16	4.41	4.50	-0.09	0
Q20	4.25	4.37	-0.12	-1
D21	4.68	4.76	-0.08	0
F22	4.45	4.66	-0.21	-1
V23	3.85	3.95	-0.10	-1
Q24	4.15	4.37	-0.22	-1
W25	4.58	4.70	-0.12	-1
L26	4.10	4.20	-0.10	-1
M27	4.35	4.52	-0.17	-1
N28	4.75	4.75	0.00	0
T29	4.40	4.35	0.05	0

Table S4. The Chemical shift index analysis of AMP-Glucagon

Residue	Real $C^{\alpha}H$ (PPM)	Reference $C^{\alpha}H$ (PPM)	Δ (Real $C^{\alpha}H$-Reference $C^{\alpha}H$) (PPM)	Index Value
S2	4.55	4.50	0.05	0
Q3	4.43	4.37	0.06	0
G4	3.98	3.97	0.01	0
T5	4.31	4.35	-0.04	0
F6	4.70	4.66	0.04	0
T7	4.29	4.35	-0.06	0
S8	4.41	4.50	-0.09	0
D9	4.69	4.76	-0.07	0
Y10	4.45	4.60	-0.15	-1
S11	4.23	4.50	-0.27	-1
L14	4.08	4.17	-0.09	0
D15	4.65	4.76	-0.11	-1
S16	4.41	4.50	-0.09	0
Q20	4.25	4.37	-0.12	-1
D21	4.68	4.76	-0.08	0
F22	4.45	4.66	-0.21	-1
V23	3.85	3.95	-0.10	-1
Q24	4.15	4.37	-0.22	-1
W25	4.58	4.70	-0.12	-1
L26	4.10	4.20	-0.10	-1
M27	4.35	4.52	-0.17	-1
N28	4.75	4.75	0.00	0
T29	4.40	4.35	0.05	0
